# Concentrically Encapsulated Dual-Enzyme Capsules for Synergistic Metabolic Disorder Redressing and Cytotoxic Intermediates Scavenging

**DOI:** 10.3390/nano12040625

**Published:** 2022-02-12

**Authors:** Chao Deng, Xianghai Li, Qianru Jin, Deliang Yi

**Affiliations:** 1 College of Chemistry & Materials Engineering, Wenzhou University, Wenzhou 325027, China; 2 Department of Chemistry, Fudan University, Shanghai 200433, China; xianghaili16@fudan.edu.cn (X.L.); jinqr03@163.com (Q.J.); yideliang@foxmail.com (D.Y.)

**Keywords:** therapeutic nanoreactor, mesoporous silica, enzyme therapy, capsule, metabolic disorder redressing

## Abstract

Enzyme therapy has important implications for the treatment of metabolic disorders and biological detoxification. It remains challenging to prepare enzymatic nanoreactors with high therapeutic efficiency and low emission of cytotoxic reaction intermediates. Here, we propose a novel strategy for the preparation of enzymes-loaded polypeptide microcapsules (EPM) with concentrically encapsulated enzymes to achieve higher cascade reaction rates and minimal emission of cytotoxic intermediates. Mesoporous silica spheres (MSS) are used as a highly porous matrix to efficiently load a therapeutic enzyme (glucose oxidase, GOx), and a layer-by-layer (LbL) assembly strategy is employed to assemble the scavenging enzyme (catalase) and polyelectrolyte multilayers on the MSS surface. After removal of the MSS, a concentrically encapsulated EPM is obtained with the therapeutic enzyme encapsulated inside the capsule, and the scavenging enzyme immobilized in the polypeptide multilayer shell. Performance of the concentrically encapsulated GOx-catalase capsules is investigated for synergistic glucose metabolism disturbance correction and cytotoxic intermediate H_2_O_2_ clearance. The results show that the EPM can simultaneously achieve 99% H_2_O_2_ clearance and doubled glucose consumption rate. This strategy can be extended to the preparation of other dual- or multi-enzyme therapeutic nanoreactors, showing great promise in the treatment of metabolic disorders.

## 1. Introduction

Enzyme therapy is attracting considerable interest in addressing metabolism disorders and biodetoxification, such as phenylketonuria, diabetes, hyperlipidemia, and hyperuricemia [1,2,3,4,5,6]. Currently reported enzyme therapy systems generally involve single types of enzymes such as uricase [7], glucose oxidase (GOx) [8,9], alcohol oxidase [10,11], superoxide dismutase [12], and phenylalanine ammonia lyase [13]. However, toxic intermediates released from the catalyzed reaction of the therapeutic enzyme (e.g., H_2_O_2_ released from the catalyzed reaction of oxidases) in a single enzyme system severely hinder the medical application [14,15,16]. In living organisms, metabolism involves an enzyme complex and physical tunnel to channel the enzymatic reaction intermediate, forming cascade reactions and avoiding the release of toxic by-products [17]. Inspired by living organisms, therapeutic nanoreactors of multienzyme systems have been proposed to redress metabolic disorders and decompose toxic intermediate molecules [18]. To improve the performance, the differently functionalized enzymes can be immobilized in a multicompartment system with a desirable spatial arrangement to minimize the free diffusion of intermediates, catalyze the cascade reactions synergistically and reduce toxic by-products release.

To date, various multienzyme nanoreactors with different spatial arrangements have been developed. These nanoreactors are mainly designed based on liposome or polymer-encapsulated enzymes including polymersomes and layer-by-layer (LbL) microcapsules [19,20,21,22]. To perform enzymatic reactions in a continuous mode, therapeutic nanoreactors are required to have an efficient membrane diffusion of substrates and products [23]. To overcome the low permeability of liposomes and polymersomes, trapping channel proteins in the polymeric bilayer or preparing permeable polymersomes with an intrinsically porous shell have been proposed [24,25]. For instance, Hest’s group has constructed a nanoreactor with positionally assembled multienzymes in porous polymersomes [26,27]. Multicompartments of liposomes, namely capsosomes, were also reported by Caruso’s group [28,29,30]. The capsosomes with concentric multicompartments could be LbL assembled by enzymes-loaded liposomes and polymer separation layers. Further, glutathione reductase or β-lactamase could be loaded in liposomal subcompartments to realize responsive release [31]. In comparison with liposomes and polymersomes, the LbL assembled microcapsules possessed more tunable permeability for the transportation of substrates. With the mediation of porous particles as sacrificial templates, LbL microcapsules can encapsulate a high payload of cargos with controllable size, permeability, degradability, and surface functionality [32,33,34]. For instance, Baumler’s group designed a multienzyme nanoreactor with magnetic biopolymer/CaCO_3_ cores and CaCO_3_ separation layers, in which the cascade reaction starts from β-glucosidase on the outer compartment to GOx in the first peripheral, and to horse radish peroxidase (HRP) in the central [35]. However, these therapeutic nanoreactors of multienzyme systems remained challenging to achieve both high therapeutic efficiency and low emission of cytotoxic intermediates.

**Scheme 1:**
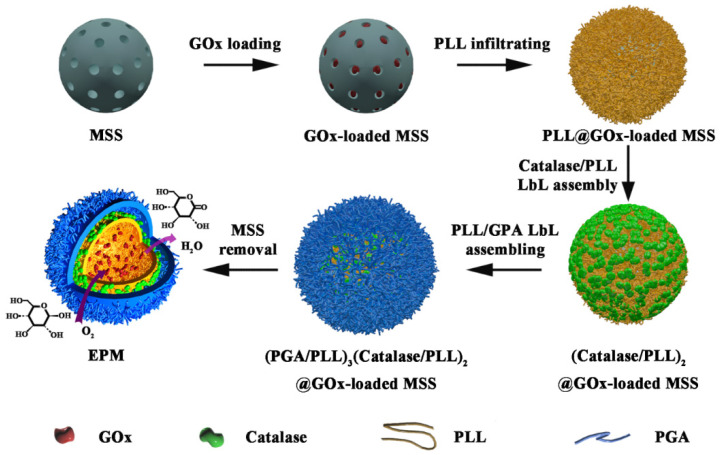
Schematic illustration of the preparation of EPM_GOx-Cat_ through MSS-mediated immobilization of the therapeutic enzyme (GOx) and concentric assembly of the scavenging catalase, followed with LbL assembly of a protecting polypeptide multilayer shell and removal of the MSS template.

Mesoporous silica spheres (MSS) have been widely used in biomedical applications due to their many distinct characteristics, such as highly tunable particle and pore size, high loading capacity, and easy surface chemical modification [36]. In this manuscript, we proposed a novel strategy to prepare enzymes-loaded polypeptide microcapsules (EPM) with concentric multicompartments through MSS-mediated enzyme loading and LbL shell assembly (Scheme 1). As a proof-of-concept, concentrically encapsulating the therapeutic enzyme (glucose oxidase, GOx) in the hollow core and scavenging enzyme (catalase, Cat) in the shell were investigated for realizing synergistic glucose metabolism disturbance correction and cytotoxic intermediate H_2_O_2_ clearance since these enzymes have been frequently employed in therapeutic nanoreactor for blood glucose redressing [10,18,37]. Owing to the concentrically encapsulated GOx and catalase and their deliberately arranged locations, the H_2_O_2_ scavenging (99% clearance rate) in the EPM system is much more efficient than previously reported multienzyme nanoreactors, such as the co-localized multienzyme nanocomplex (about 85%) [10] and the supramolecular hydrogel (about 88%) [37]. The concentrically compartmented EPM holds enormous promise in developing more multienzyme therapeutic nanoreactors for addressing metabolic disorders and scavenging toxic intermediates.

## 2. Materials and Methods

### 2.1. Materials

Catalase from bovine liver (C100, 45,573 U mg^−1^), glucose oxidase from *Aspergillus niger* (Type X-S, 127 U mg^−1^), acetic acid (HAc), 4-morpholineethan-esulfonic acid (MES hydrate), poly(acrylic acid) (PAA, average M_w_ ~250 kDa, 35 wt.% solution in H_2_O), tetraethyl orthosilicate (TEOS), poly-L-lysine hydrobromide (PLL, M_w_ 30–70 kDa or small molecular weight PLL, s-PLL, Mw 1–5 kDa), poly-L-glutamic acid sodium salt (PGA, M_w_ 15–50 kDa), 4-morpholineethan-esulfonic acid (MES hydrate), glutaraldehyde (GA, 50% in H_2_O), 3-aminopropyl triethoxysilane (APTS), hexadecyltrimethylammonium bromide (CTAB), 3,3’,5,5’-tetramethylbenzidine (TMB), 7-(diethylamino)coumarin-3-carboxylic acid N-succinimidyl ester (DCCA), sulforhodamine B acid chloride (SRB), and fluorescein isothiocyanate isomer I (FITC) were purchased from Sigma-Aldrich (Shanghai, China). Horseradish peroxidase (HRP, 200 U mg^−1^) and 1-ethyl-3-(3-dimethylaminopropyl) carbodiimide hydrochloride (EDC) were purchased from Aladdin Reagent Company (Shanghai, China). All chemicals were of at least analytical grade and used without further purification. Fluorochrome-labeled enzymes and PLL (GOx labeled with green FITC, catalase labeled with blue DCCA, and PLL labeled with red SRB) were prepared by adding 50 μL of dye (10 mg mL^−1^) to 1.0 mL of enzyme or PLL solution (10 mg mL^−1^) in 0.1 M sodium bicarbonate buffer at pH 9.0. After stirring at room temperature for 1 h, the mixture was sufficiently dialyzed against deionized water to remove free dyes.

### 2.2. Preparation of EPM

Briefly, the preparation of GOx-loaded EPM (EPM_GOx_) included three steps. (1) GOx loading: 5.0 mg of amine-modified MSS (dispersed in ethanol, supporting information) was washed with HAc buffer (50 mM, pH 5.0) twice to remove ethanol. Then 2.0 mL of GOx solution (1.0 mg mL^−1^) was added to the MSS suspension. After incubation at room temperature for 12 h, GOx-loaded MSS were collected by centrifugation (1500 g, 5 min). To crosslink the amino groups in GOx with the amine-modified MSS, 1.0 mL of fresh GA solution (0.2 wt.%, in HAc buffer, pH 5) was added to the GOx-loaded MSS suspension and incubated at room temperature for 1 h. After that, the particles were centrifuged and washed with HAc buffer twice. (2) PLL infiltrating: 1 mL of s-PLL (5 mg mL^−1^ in PBS buffer, pH 7.4) was used to infiltrate into the porous particles for 12 h. After the infiltration of s-PLL and removal of excess s-PLL, 2.0 mL of freshly prepared EDC solution (4 mg mL^−1^ in MES buffer, pH 5.5) was added to crosslink the residual carboxyl groups on GOx and amino groups on s-PLL. After 2 h of incubation, the obtained materials were washed with MES buffer twice. (3) LbL shell assembling: Three PLL/PGA bilayers were deposited on the MSS surface by mixing 2.0 mL of alternative PGA/PLL (1 mg mL^−1^ in MES buffer) and MSS for 10 min. Twice washing with MES buffer was applied to remove excess polypeptide after each step of polypeptide deposition. After that, EDC was added to crosslink the assembled enzymes and polypeptides. The EPM_GOx_ was obtained after removal of the MSS template with 0.3 mL of HF/NH_4_F solution (2 M HF: 8 M NH_4_F) for 1 min. The loading and leaching of Gox during EPM_Gox_ preparation were monitored by UV-vis spectrophotometry at the band of 276 nm (Figure S1). The concentrically encapsulated dual enzymes capsule (i.e., EPM_Gox-Cat_) was prepared by the same procedure for EPM_Gox_ preparation except that the second enzyme (catalase) was sequentially assembled on the Gox-loaded MSSs before the PLL/PGA multilayer coating.

### 2.3. Enzyme Activity Assay

Enzymatic activity of Gox was monitored by combining HRP and TMB-based color reactions. Briefly, 10 μL of sample was added to 2.0 mL of HAc buffer (pH 4.0) containing 15 ng of HRP and 10 μL of TMB (10 mg mL^−1^). The color reaction was initiated by adding 20 μL of 10 mM glucose solution at 37 °C. After 20 min, 50 μL of H_2_SO_4_ solution (2.0 M) was added to end the reaction and the UV-vis absorbance at 450 nm was recorded. Enzymatic activity of catalase was monitored by adding 10 μL of sample to 2.0 mL of 20 mM H_2_O_2_ solution in 50 mM PB buffer (pH 7.0) and the decomposition of H_2_O_2_ with time was measured spectrophotometrically at 240 nm. Catalase active unit was defined as the amount of catalase which decomposed 1 μmol of H_2_O_2_ per min at pH 7.0 at 25 °C. The changes of glucose and H_2_O_2_ concentration catalyzed by EPM in sealed glucose solution were monitored by a combining GOx-HRP color reaction in a 96-well microplate. Firstly, 20 μL of EPM (~8.6 μg of GOx) was added into 1.0 mL of glucose solution (10 mM) sealed with 1 mL of n-hexane in a water bath at 37 °C. Secondly, 10 μL of sample was extracted from glucose solution at a certain time intervals and added into a 10 μL of 2.0 M H_2_SO_4_ solution to deactivate the EPM. Finally, distinguishing measuring samples were added into 96-well microplates with 200 μL of HAc buffer (pH 6.0), 10 μL of GOx (1.0 mg mL^−1^, excluded in the case of H_2_O_2_ assay), HRP (0.1 mg mL^−1^), and TMB (10 mg mL^−1^) solution. After incubating at 37 °C for 30 min, 10 μL of H_2_SO_4_ solution (2.0 M) was added to each well and the absorbance at 450 nm was recorded. The concentration of H_2_O_2_ was calculated based on the color reaction in absence of GOx compared to standard H_2_O_2_, while that of glucose was calculated by the difference value in the presence and absence of GOx.

### 2.4. Blood Glucose Reduction Assay

To assess the efficiency of EPM_GOx-Cat_ on catalytic reduction of blood glucose, whole blood was extracted from an adult mouse with the addition of heparin and extra glucose. Then, 0.5, 1.0, or 2.0 μL of EPM_GOx-Cat_ suspension was added into 200 μL of whole blood in a water bath at 37 °C. Then, 10 μL of blood was withdrawn from the reaction system at fixed time intervals for immediate detection of the blood glucose concentration.

### 2.5. Cell Viability Assay

HeLa cells were seeded into a 96-well plate (~10^4^ cells per well) containing 200 µL of DMEM media (high level of glucose) at 37 °C in 5% CO_2_. After 12 h of incubation, 1.0 µL of EPM, EPM_GOx_, or EPM_GOx-Cat_ suspension was added, and the sample was further incubated for 3 h before the 3-(4,5-dimethylthiazol-2-yl)-2,5-diphenyltetrazolium bromide (MTT) assay to test the cell viability.

### 2.6. Characterization

Transmission electron microscope (TEM) images of EPM were collected from the Hitachi H-600 microscope (Tokyo, Japan). The high-resolution TEM images of MSS were obtained from Tecnai G2 F20 S-Twin (Hillsboro, OR, USA). Scanning electron microscope (SEM) images were collected from a field emission SEM (Zeiss Ultra 55, Oberkochen, Germany). Fourier-transform infrared (FTIR) spectra were measured on a Nicolet 6700 Spectrometer (Thermo Fisher Scientific, Waltham, MA, USA). The fluorescence images were obtained from a Leica DM4000 B microscope (Wetzlar, HE, Germany). The UV-vis spectra were recorded on a Shimadzu, UV-2600 spectrophotometer (Shimadzu, Kyoto, Japan). The blood glucose concentration was monitored by a one-touch blood glucose meter (Lifescan Inc., Seattle, WA, USA).

## 3. Results and Discussion

**Figure 1:**
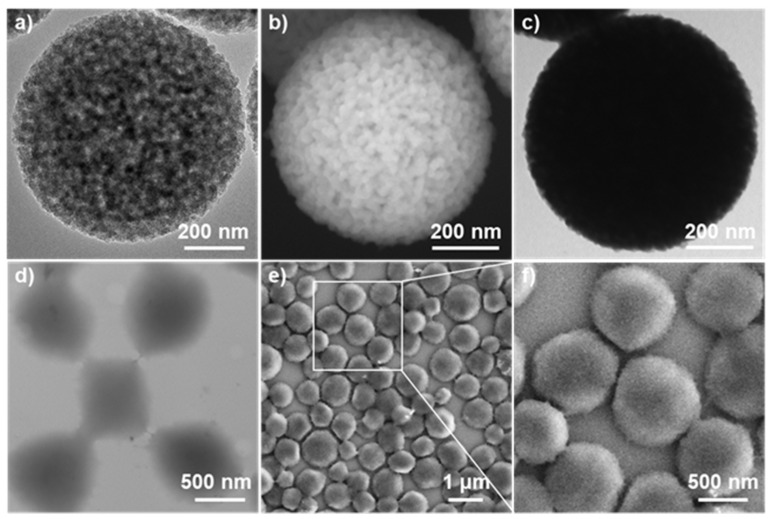
TEM image (**a**) and SEM image (**b**) of the APTS-modified MSS. TEM images of the (PGA/PLL)_3_(Catalase/PLL)_2_@GOx-loaded MSS (**c**). TEM image (**d**) and SEM images (**e**,**f**) of the EPM_GOx-Cat_.

The MSS with an average particle size of 800 nm was synthesized according to a method reported earlier [38]. High resolution TEM and SEM images revealed that the MSS has a dual pore distribution (~3 nm and ~20 nm) (Figure 1a,b). GOx, molecular size of 6–7 nm, was used as the therapeutic enzyme for glucose oxidation. Hence, the larger mesopores in the MSS are important for achieving high content of GOx loading inside the particles. To prepare EPM with concentrically encapsulated dual enzymes, catalase and PLL was sequentially assembled on the GOx-loaded MSSs. By using this strategy, various enzymes with tunable content could be facilely assembled on the particle surface to construct concentric compartments. (Scheme 1). It is worth noting that the MSS has dual roles, one is to effectively load enzymes inside the highly porous particle, and the other is to provide a spherical template for LbL assembling the polymeric capsule. The enzyme capsules would not form without using the MSS template, since aggregated enzyme and polyelectrolyte complex would be generated when the negatively charged enzymes were mixed with the positively charged PLL. The TEM images indicated a significantly lower density of the particles due to the removal of the inorganic MSS template (Figure 1c,d). The SEM images of EPMGOx-Cat showed a bulging morphology (Figure 1e,f), primarily due to their high enzyme loading and structural robustness of the EPM.

As GOx has an isoelectric point (IEP) of 4.2, the amino-modification of the MSS is essential to obtain a high enzyme loading through the electrostatic interaction of the negatively-charged GOx and the positively-charged MSS surface. The loading of GOx in the MSS particles could be adjusted by changing the mass ratio of MSS to GOx in the incubation solution. When the MSS/GOx mass ratio was set at 2.5, 250 mg of GOx could be loaded in one gram of MSS particles (i.e., 25 wt.% GOx loading), which corresponded to an enzyme immobilization rate of 62.5%. The GOx loading could be increased to 35 wt.% when the MSS/GOx mass ratio was set at 1, however, the enzyme immobilization rate was decreased to ~36%. Thereafter, the mass ratio of MSS to GOx was fixed at 2.5 in following experiments.

**Figure 2:**
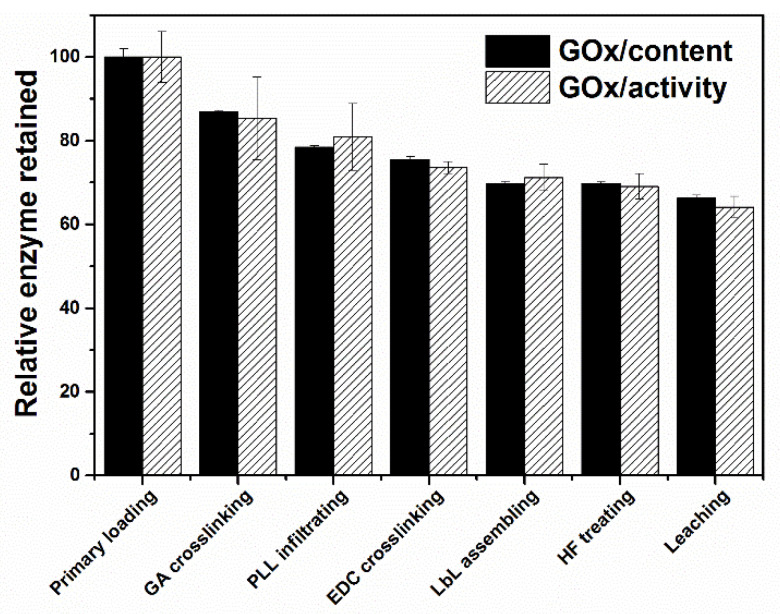
Variation of GOx content and activity after each step of the process. The loading and leaching contents of GOx were determined by UV-vis detection of supernatant solution. Data were obtained based on the average of three batches of EPM preparation.

As the GOx molecules were adsorbed into the mesopores through electrostatic interaction, changing of the solution environments (e.g., pH, salt concentration, or in the presence of competitive adsorption molecules) could cause leakage of the physically adsorbed enzyme molecules. It was found that up to 90% of the loaded GOx could be released during the procedure to assemble a PLL/PGA multilayer shell on the MSS to encapsulate the loaded enzyme. To minimize the leakage of loaded GOx, 0.2 wt.% GA solution was used to crosslink the enzyme molecules to form a covalently connected enzyme network inside the mesopores. There is only ca. 20% of GOx released during the GA crosslinking and the subsequent PLL infiltrating steps (Figure 2).

GOx was immobilized steadily in the MSS after GA crosslinking, however, 80% of the immobilized enzymes would be released provided the MSS template was removed. Inspired by previous work that enzymes could be stably immobilized inside nanoporous polymer particles by bridging proteins with infiltrated polymer [39,40], we combined polymer infiltrating and LbL-based multienzyme assembling with a crosslinking process to couple the amine groups and carboxyl groups under the catalyst of EDC. In this process, the s-PLL with less steric effect was used to infiltrate into the GOx-loaded mesopores and bridging GOx (Scheme 1). Then, a protective PLL/PGA bilayer shell was LbL assembled on the particle surface to encapsulate the enzymes. Finally, the MSS template was dissolved by adding an HF buffer. About 97% of the catalytic activity of enzymes was retained after HF treatment, implying the successful enzyme immobilization through the s-PLL infiltrating and EDC crosslinking process. The stable immobilization of GOx in the crosslinked PLL/PGA multilayer capsule could be further demonstrated from the leaching tests, in which 93–95% of GOx activity was retained in the capsules after shaking the EPM_GOx_ in PBS at 37 °C for 24 h (Figure 2). The final product, EPM_GOx-Cat_, contained about 430 μg mL^−1^ of GOx (55 U mL^−1^) and 58 μg mL^−1^ of catalase (2651 U mL^−1^). In a similar leaching test in PBS buffer, about 98% of catalase was retained in EPM_GOx-Cat_. The enzyme retention rate in the capsules was directly related to the structural stability of EPM. The high retaining rate of GOx and catalase in the EPM clearly suggested the structural stability of the crosslinked EPM.

**Figure 3:**
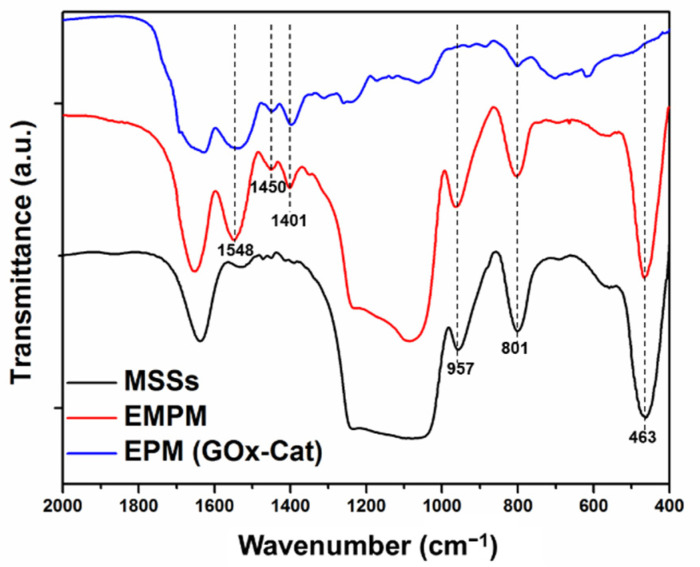
FTIR spectra of MSSs, EMPM, and EPM_GOx-Cat_.

To monitor component changes of the samples, FTIR spectra of relative intermediates including MSSs, (PGA/PLL)_3_(Catalase/PLL)_2_@GOx-loaded MSSs (EMPM), and EPM_GOx-Cat_ were detected (Figure 3). The FTIR spectra of MSSs and EMPM both exhibited peaks at the bands attributed to Si−O−Si bending vibration (463 cm^−1^), Si−O−Si symmetric stretching (801 cm^−1^), surface Si−OH groups (957 cm^−1^), and Si−O−Si asymmetric stretching (1000–1200 cm^−1^) [41]. These peaks were totally disappeared in the EPM, indicating the efficient removal of the MSS template. The absorption band at 1638–1653 cm^−1^ displayed in the MSSs and EMPM was mainly attributed to the overlap of the amino group (1630 cm^−1^) and water molecules (1652 cm^−1^) [41,42]. After enzyme loading and PLL/PGA polypeptide multilayer assembling, the FTIR spectra of EMPM showed a series of additional peaks that could be attributed to COO^−^ symmetric stretching vibration (1401 cm^−1^), CH_3_/CH_2_ in-plane bending vibration (1450 cm^−1^), COO^−^ antisymmetric stretching vibration and NH_3_^+^ symmetric stretching vibration (1548 cm^−1^) [23]. These peaks were retained in the EPM, suggesting that the enzymes and polypeptides were well preserved after the removal of the MSS template.

**Figure 4:**
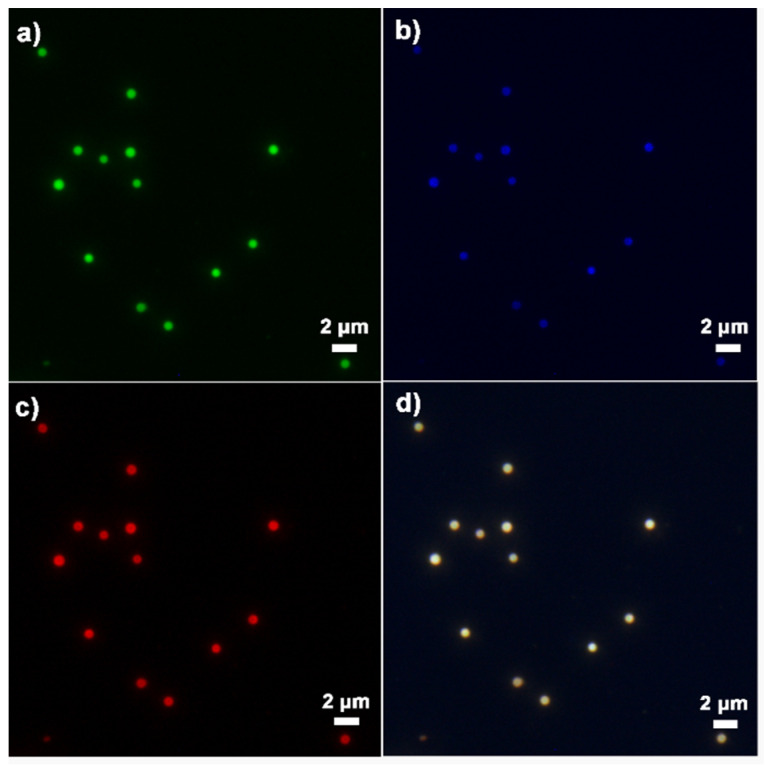
Fluorescence microscope images of EPM_GOx-Cat_ to visualize the loading of green dye FITC-labeled GOx (**a**), blue dye DCCA-labeled catalase (**b**), and red dye SRB-labeled PLL (**c**) in the capsules, and their merged image (**d**).

Homogeneity of the enzyme-loaded EPM was studied via fluorescence microscopy images. For distinguishing different species in the EPM, three fluorescent dyes with distinguishing excitation and emission wavelengths, including FITC (green), DCCA (blue), and SRB (red), were used to label GOx, catalase, and PLL, respectively. The brightly colored particles seen in the images (Figure 4a–c) reflected the homogeneous loading of each component in the capsules. The fluorescent microscope images also suggested the monodispersity of the EPM_GOx-Cat_ in solution. The merged fluorescent image shown in Figure 4d clearly demonstrates the colocalization of GOx, catalase, and PLL in EPM_GOx-Cat_.

**Figure 5:**
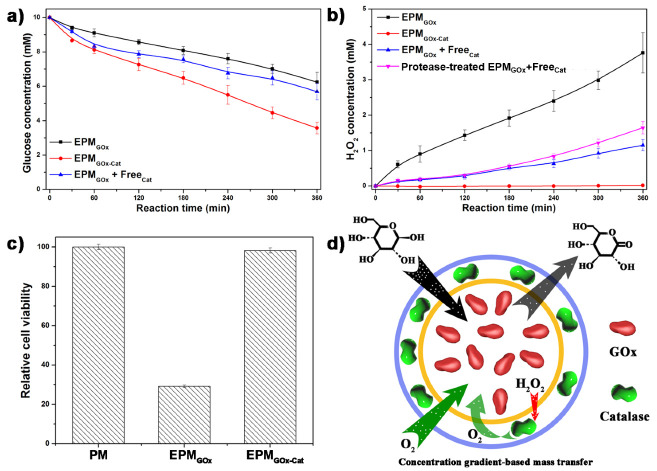
(**a**) Synergistic effect of the EPM on glucose redressing. Cytotoxic intermediate H_2_O_2_ scavenging properties of EPM as exhibited either in the form of (**b**) H_2_O_2_ concentration in PBS or (**c**) cell viability in HeLa culture media. The concentration of glucose (**a**) and H_2_O_2_ (**b**) were monitored in three independent glucose oxidation tests for an average measurement. (**d**) The schematic illustration of H_2_O_2_ scavenging and synergistic function based on concentric compartment of GOx and catalase in the EPM_GOx-Cat_. The concentration gradient and diffusion direction of substances were indicated by the arrows.

Roles of the concentric compartment on scavenging the toxic reaction intermediate H_2_O_2_ and cycling the cascade reaction were studied. To this purpose, EPM_GOx-Cat_ was used to oxidize glucose in PBS solution in an enclosed environment. The concentration of glucose decreased by EPM_GOx_ and EPM_GOx-Cat_ showed approximately linear reduction (Figure 5a), with a total glucose consumption of 3.9 mM and 7.2 mM, respectively, in 6 h. Importantly, the reaction rates ofEPM_GOx_ (10.8 μM min^−1^) and EPM_GOx-Cat_ (20.0 μM min ^−1^) were significantly different, exhibiting a synergistic effect of EPM_GOx-Cat_. Catalase catalyzed the decomposition of cytotoxic intermediates H_2_O_2_ and generates O_2_, which could be further reused in the GOx catalyzed glucose oxidation reaction. The cascade reaction was performed circularly based on Equations (1) and (2), in which the cycle ratio could be significantly enhanced by the rapid H_2_O_2_ scavenging and the concentrically arranged bioreactor compartments in a microenvironment.

(1) $$\mathrm{glucose}+\;O_{2}\;\overset{\mathrm{GOx}}{\to }\;\mathrm{gluconolactone}\;+\;H_{2}O_{2}$$

(2) $$H_{2}O_{2}\;\overset{\mathrm{Catalase}}{\to }\;H_{2}O\;+\;\frac{1}{2}\;O_{2}$$

H_2_O_2_ generated from glucose decomposition by EPM_GOx_ exhibited a linear increase reaching up to 4 mM in 6 h (Figure 5b). On contrary, only 74 μM H_2_O_2_ was found in surrounding media after decomposing 7.2 mM glucose by EPM_GOx-Cat_ under the same reaction conditions. In other words, 99% H_2_O_2_ generated in the EPM_GOx-Cat_ system was successfully eliminated by downstream scavenging enzyme-catalase. In the case of the protease pre-treated EPMGox, the content of the H_2_O_2_ produced was very close to that of H_2_O_2_ generated from the untreated EPMGox, demonstrating that the capsule can protect the loaded enzymes from protease degradation. To the best of our knowledge, the H_2_O_2_ scavenging in the EPM_GOx-Cat_ system is much more efficient than other multienzyme nanoreactors, such as co-localized multienzyme nanocomplex (about 85%) [10] or supramolecular hydrogel (about 88%) [37]. The culture media of HeLa cells was further used to demonstrate the cytotoxicity of H_2_O_2_ generated during the glucose oxidation (Figure 5c). After 3 h of incubation, the generated H_2_O_2_ from EPM_GOx_ resulted in 71% of mortality, while that from EPM_GOx-Cat_ resulted in only 2% of mortality, very close to the peptide-multilayered microcapsules (PM), in which no H_2_O_2_ was generated.

Although catalase is commonly considered as an efficient H_2_O_2_ scavenging enzyme for therapeutic purposes, the synergistic effect of GOx and catalase on nanoreactor design has been rarely reported [10,37,43]. In living organisms, a physical tunnel of enzyme complex is developed to limit and channel the intermediates essentially [17]. It must be pointed out that current co-loading or multicompartments of multienzymes in a nanoreactor fail to limit the free diffusion of reaction intermediates. In the case of a GOx and catalase dual-enzyme system, the generated H_2_O_2_ and regenerative O_2_ will keep free diffusion and only part of these is involved in the cascade enzymatic reaction, resulting in compromised H_2_O_2_ scavenging. Herein, we designed the concentric compartment of EPM_GOx-Cat_ to limit the release of toxic H_2_O_2_ and further utilized it as a useful intermediate for O_2_ generation. On the whole, during the enzymatic reaction, all substrates and products form a certain concentration gradient (Figure 5d), imposing mass transfer restriction on the reaction substrates (outside-in approach) and the products (inside-out approach). The oxidation of one glucose molecule catalyzed by GOx consumes one O_2_ and generates one H_2_O_2_ molecule, which could be decomposed by catalase to produce half O_2_ for recycling use. The high-efficiency clearance of H_2_O_2_ was achieved by arranging catalase on the downstream position of the H_2_O_2_ concentration gradient (Figure 5b). Significantly, the regenerated O_2_ from catalase decomposition of H_2_O_2_ will diffuse inward to EPM due to the O_2_ partial pressure gradient (Figure 5d). The iterative cycle of H_2_O_2_ doubled the utilization ratio of original O_2_ from 50% to 100% and further enhanced the oxidation rate of glucose compared with EPM_GOx_ in the same reaction condition (Figure 5a). The low emission of H_2_O_2_ and synergistic effects were simultaneously achieved by concentric encapsulation of dual enzymes. Furthermore, the roles of the concentric compartment and created microenvironment are not influenced by diluting or fast diffusing liquid such as blood, which brought extra potentials for therapeutic nanoreactor application.

**Figure 6:**
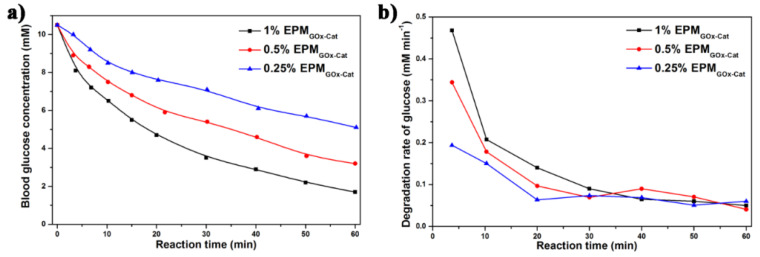
EPM_GOx-Cat_ dose-dependent redressing of blood glucose. (**a**) Blood glucose concen-tration profile and (**b**) degradation rate profile of glucose with diffirent ratio of EPM_GOx-Cat_.

The therapeutic application of nanoreactors on metabolic disorder redressing was demonstrated through fast blood glucose oxidation by EPM_GOx-Cat_. Different doses of EPM_GOx-Cat_ were added into mouse whole blood to decrease blood glucose. As shown in Figure 6, the initial glucose consumption rate of EPM_GOx-Cat_ in blood was about 400 μM min^−1^, much higher than that of 20 μM min^−1^ obtained in PBS solution (Figure 5a). This enhancement was primarily ascribed to the increase of the total blood oxygen capacity induced by hemoglobin in red blood cells. Meanwhile, the glucose oxidation rate by EPM was dose-dependent. For instance, the EPM_GOx-Cat_ with a dosage of 1%, 0.5%, and 0.25% (*v*/*v*) required a treatment time of around 15, 30, and 60 min, respectively, to redress the high blood glucose (10.5 mM) to normal blood glucose (~5 mM).

It must be pointed out that over-consumption of blood glucose will cause hypoglycemia, which is dangerous for health. Even taking account of fast glucose supplement from hepatic glycogen, therapeutic nanoreactor should also consider the problem of over-consumption of glucose or other biomolecules. The ratio of EPM_GOx-Cat_ and blood could be adjusted to avoid the over-consumption of blood glucose, as the dose of EPM_GOx-Cat_ was directly proportional to the amount of glucose consumption (Figure 6). Interestingly, the glucose oxidation rate of all EPM dosages decreased to a similar level of 0.04–0.06 mM min^−1^, when the glucose concentration is approaching the normal blood glucose. For instance, the glucose consumption rate of 1% EPM sample declined around 9 times (from 470 μM min^−1^ to 50 μM min^−1^) in 60 min, while those of 0.5% and 0.25% EPM declined around 7 times and 4 times, respectively. The reduction of reaction rate was mainly caused by the decreased concentration of substrates. This typical enzymatic reaction feature was further amplified by concentration-dependent diffusion of substances in EPM.

## 4. Conclusions

In this work, we have designed and fabricated potent therapeutic capsules by loading GOx inside MSS, assembly of catalase on the shell, and subsequent encapsulating the loaded enzymes with a PLL/PGA multilayer shell. After crosslinking, GOx and catalase were stably trapped inside the concentric multicompartments of EPM with uniform distribution and high enzyme loading. Owing to the concentrically encapsulated GOx and catalase and their deliberately arranged locations, 1% (*v*/*v*) EPM_GOx-Cat_ can potently redress hyperglycemia from 10.5 to 5 mM in 15 min with an initial glucose consumption rate of 400 μM min^−1^ in mouse whole blood. Meanwhile, 99% of the cytotoxic reaction intermediates (H_2_O_2_) scavenging ratio and double glucose consumption rate were realized. The highly efficient and concentrically compartmented features of EPM holds enormous promise in developing more multienzyme therapeutic nanoreactors for addressing metabolic disorders and scavenging toxic intermediates.

## Data Availability

Not applicable.

## Supplementary Materials

The following supporting information can be downloaded at: https://www.mdpi.com/article/10.3390/nano12040625/s1, Figure S1: UV-Vis spectra of GOx at different concentrations (**a**) and the standard calibration curve by using the absorbance at 276 nm (**b**).

## Abbreviations

EPM	enzymes-loaded polypeptide microcapsules
GOx	glucose oxidase
LbL	layer-by-layer
PGA	poly-L-glutamic acid
PLL	poly-L-lysine
MSS	mesoporous silica sphere
