# Supplementary material for: Concentrically Encapsulated Dual-Enzyme Capsules for Synergistic Metabolic Disorder Redressing and Cytotoxic Intermediates Scavenging
Source: Nanomaterials (Basel). 2022 Feb 12;12(4):625. doi: 10.3390/nano12040625 (PMC8878113; doi:10.3390/nano12040625)
Supplement: Supplementary file 1 [file nanomaterials-12-00625-s001.zip › nanomaterials-1578141-supplementary.pdf]

nanomaterials

Supplementary Materials

Concentrically Encapsulated Dual-Enzyme Capsules for Synergistic Metabolic Disorder Redressing and Cytotoxic Intermediates Scavenging

Chao Deng [1,*,†], Xianghai Li [2,†], Qianru Jin [2] and Deliang Yi [2]

1 College of Chemistry & Materials Engineering, Wenzhou University, Wenzhou 325027, China
2 Department of Chemistry, Fudan University, Shanghai 200433, China; xianghaili16@fudan.edu.cn (X.L.); jinqr03@163.com (Q.J.); yideliang@foxmail.com (D.Y.)
* Correspondence: dengchao@wzu.edu.cn
† Contributed equally to this work.

1. Synthesis of MSSs

In a typical synthesis, 0.55 g hexadecyltrimethylammonium bromide (CTAB) was completely dissolved in 25.0 ml deionized water with stirring, and 3 g poly(acrylic acid) (PAA) (25 wt% solution) was added with vigorous stirring at room temperature to obtain a clear solution. Next, 2.0 g of ammonia (25%) was added to the above solution with vigorous stirring, pH 10–11. The solution immediately turned into a milky suspension due to the formation of the PAA/CTAB complex. After a further 20 minutes of stirring, 2.08 g of tetraethylsiloxane (TEOS) was added to the above solution. After stirring for 15 minutes, transfer the mixture to the autoclave and place it at room temperature (R.T.) or in an oven at 80 °C for 48 hours. The white final product was centrifuged, washed with deionized water, and dried at 60 °C. Organo-templates were removed by calcination at 550 °C for 6 h. Calcined mesoporous silica sphere (MSS) was modified with aminopropyl group. 10.0 mg of calcined MSS was dispersed in 1.4 mL of ethanol, then 0.25 mL of APTES and 60 µL of ammonia solution (NH_3 content of 25 wt%) were sequentially added, Fand the mixture was shaken at room temperature for 8 h. The product was isolated from the mixture by centrifugation and washed 3 times with ethanol and water, and the final product was stored in ethanol (10 mg mL⁻¹).

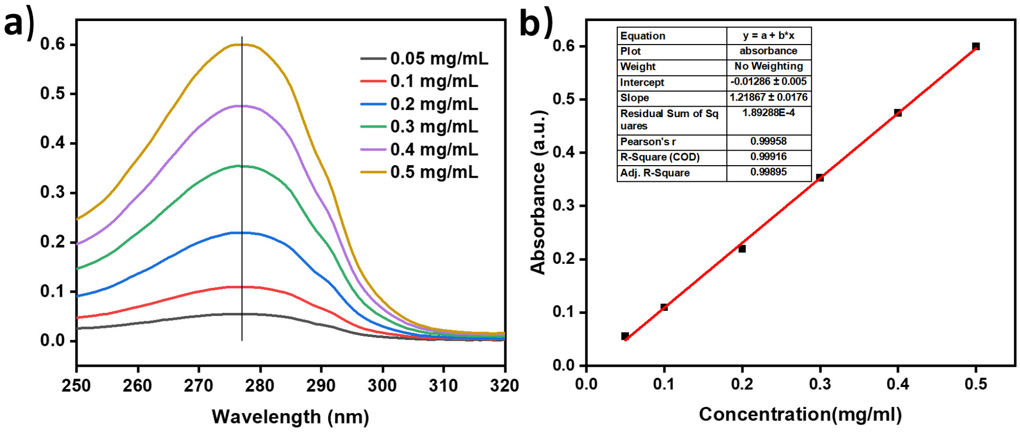

Figure S1. UV-Vis spectra of GOx at different concentrations (**a**) and the standard calibration curve by using the absorbance at 276 nm (**b**).
